# The Usefulness of MS^3^ to Confirm Poisoning on the Example of Dog Poisoning with Strychnine

**DOI:** 10.3390/molecules24203765

**Published:** 2019-10-19

**Authors:** Tomasz Śniegocki, Bartosz Sell, Andrzej Posyniak

**Affiliations:** Department of Pharmacology and Toxicology, National Veterinary Research Institute, 24-100 Pulawy, Poland; bartosz.sell@piwet.pulawy.pl (B.S.); aposyn@piwet.pulawy.pl (A.P.)

**Keywords:** strychnine, forensic veterinary toxicology, poisoning, QuEChERS, HPLC-MS^3^

## Abstract

Strychnine is an alkaloid with strong toxic properties. Poisoning results in muscular contractions and death through asphyxiation. Intentional or accidental poisonings with strychnine occur mainly in small animals, especially dogs and occasionally cats. Strychnine can be detected in the liver or stomach contents. Unfortunately, the determination of strychnine in these matrices, especially in postmortem examination, is subject to a significant matrix effect that makes it difficult to confirm the presence of the substance being determined. Therefore, we developed a new liquid chromatography method combined with mass spectrometry. One-gram homogenized samples were extracted and partitioned after adding acetonitrile and 5-mol solution of ammonium acetate. After extraction, the samples were analyzed using high-pressure liquid chromatography-MS/MS/MS. The results of validation fulfil the requirement of the confirmatory criteria according to SANTE/11945/2015 regarding apparent recoveries (98.97% to 104.0%), repeatability (2.9%–4.1%), and within-laboratory reproducibility (3.3%–4.6%). The method can be successfully applied to confirm strychnine poisoning cases.

## 1. Introduction

Strychnine is an alkaloid with extremely toxic properties. In the past it was used as a rodenticide against mice and rats, and in medicine as a laxative, appetizer, and stimulant of the central nerve system [1,2]. Strychnine is highly toxic after being used orally, through inhalation or in absorption through the mucous membranes of the mouth or eyes. It is quickly absorbed after oral administration, metabolized by the hepatic enzymes, and readily distributed to many different tissues [1,3]. Poisoning results in muscular contractions and death through asphyxiation [1,3]. Acute toxicity expressed as half-lethal dose (LD_50_) in dogs, cattle, horses, and pigs ranges between 0.5 and 1 mg/kg, and 2 mg/kg for cats, respectively [3]. Currently, in European Union countries there are no approved products containing strychnine as an active compound. For many years, strychnine has been used as an appetite stimulant due to its bitter taste, which stimulates gastric and salivary secretion. Intentional or accidental poisonings with strychnine occur mainly in small animals, especially dogs and occasionally cats, and rarely in farm animals. From animals, for the purposes of toxicological examinations, the most frequently used matrices are stomach contents, blood, and liver, whereas from environmental samples they are baits and feeds [4,5,6,7,8,9,10,11,12]. Cases of strychnine poisoning are usually acute or hyperacute. In such cases, the toxin most often has reached high concentrations in the stomach contents, but it is also present in the liver [12]. The liver samples are carefully chosen based on the expected concentrations and analytical techniques used for examination, as a best choice to control their presence in poisoned animals. This kind of sample is complex and can contain different interferences (i.e., liver from different animal classes like omnivores, carnivores, and herbivores). Additionally, the determination of strychnine in liver matrices—especially postmortem—can cause a significant matrix effect, which makes it difficult to confirm the presence of the substance being determined. That is why we decided to create a new method based on QuEChERS with the combination of liquid chromatography coupled with mass spectrometry (HPLC–MS^3^). QuEChERS is a method commonly used in pesticide determination, which we also successfully use to determine toxic substances like rodenticides, carbamate and organophosphorus pesticides, coccidiostats, and mycotoxins [12]. Additionally, the combination of this method with HPLC–MS^3^ made it easier to confirm strychnine in liver matrices. The MS^3^ technique compared to MS^2^ techniques can significantly improve the selectivity of the method [13,14,15,16,17]. In addition, the method’s repeatability is better, chromatographic interference is lower, and a matrix effect was observed [16,17]. The new HPLC-MS^3^ was validated, confirming that this procedure fulfils the requirements of the confirmatory criteria according to SANTE/11945/2015 [18].

## 2. Results and Discussion

**Figure 1:**
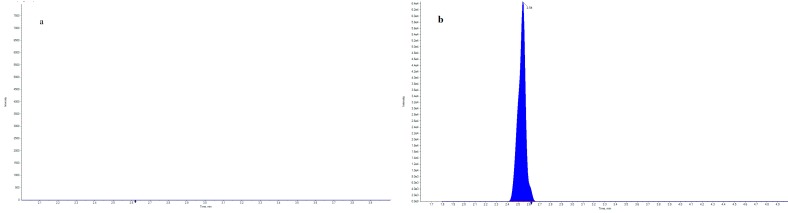
Example chromatograms of (**a**) blank liver sample and (**b**) liver containing strychnine residues at a concentration of 150 µg/kg.

**Figure 2:**
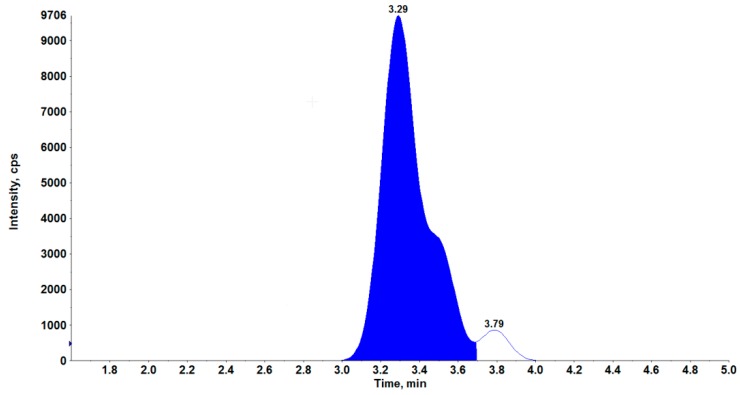
Example chromatogram of liver containing strychnine residues at the limit of quantification (LOQ).

This is the first method published for the confirmation of strychnine in dog liver based on QuEChERS combined with an HPLC-MS^3^ procedure. To achieve better sample purification—especially in liver—instead of previously used sodium acetate salt [12] we used a 5 molar solution of ammonium acetate to provide an additional water-based layer with a high concentration of impurities in each sample at pH = 7.8. Ammonia was used to stabilize the pH of the buffer at a level similar to that of sodium acetate solution, which had no influence on the validation parameters. Based on our previous experience [12] we intended to use positive ionization. The following parameters were optimized by direct flow infusion: source temperature, desolvation gas temperature, desolvation gas, capillary voltage, cone voltage, collision energy, second precursor ion, excitation energy, scan window width, entrance potential, and declustering potential. Separation of strychnine is generally performed by LC using reversed-phase 100 mm C18 columns with a mobile phase of methanol-20mM ammonium formate-formic acid [2]. We decided to use a shorter column with C8 reversed-phase for a better peak shape. Because the composition of the mobile phase in the previously described method gave a symmetric peak shape and minimized the matrix effect [12], we decided to use this mobile phase (0.5% isopropanol in 0.1% acetic acid in water and 5% isopropanol in ethanol), but with a gradient and column modification. Example chromatograms of a blank liver sample and another liver sample containing strychnine residues at a concentration of 150 µg/kg are shown in Figure 1 and at the limit of quantification (LOQ) in liver matrix in Figure 2.

**Figure 3:**
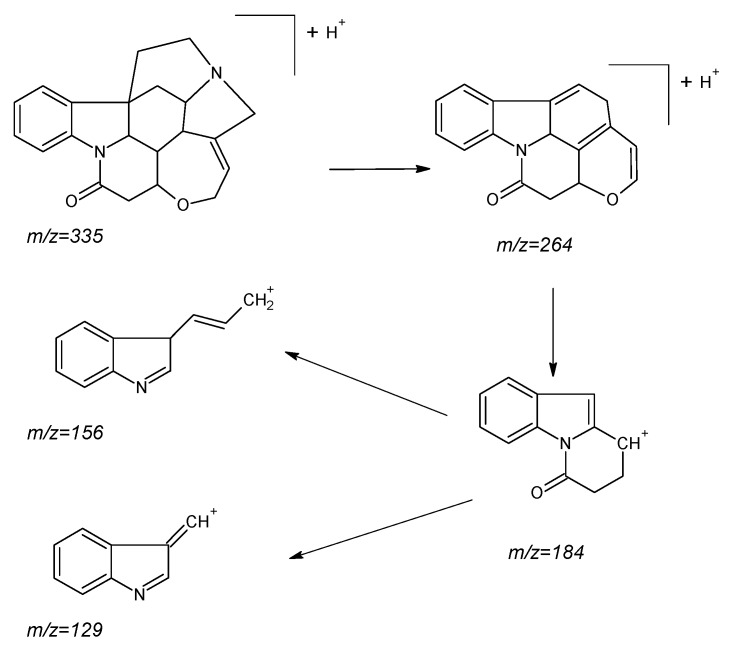
The proposed fragmentation path for strychnine.

**Figure 4:**
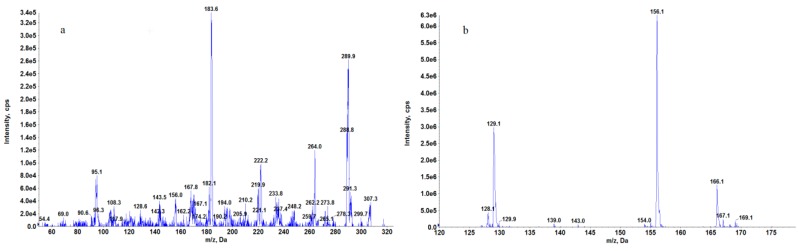
(**a**) MS^2^ and (**b**) MS^3^ spectra of strychnine in liver matrix.

The main problem in the determination of strychnine in the liver collected from dead animals—especially in cases where this organ has already partially decayed—is the possible high matrix effect. That is why we decided to create an HPLC-MS^3^ method for the determination of strychnine in the liver obtained from dead animals. The use of this technique provides increased selectivity by selecting the second-generation fragment as an analyte signal, which results in better method repeatability, reproducibility, and better signal-to-noise ratio [13,14,15,16,17]. Chen et al. also determined strychnine with MS^3^ and obtained very good results, but in this case the ion trap was used and did not allow such low concentrations to be achieved as were achieved in our method [19]. In addition, the authors do not provide the values of repeatability and reproducibility of their method. Our method produced comparable data versus multiple reactions monitoring (MRM) for the quantifier signal, but much better accuracy and sensitivity for the qualifier signal in liver matrix. The proposed fragmentation path for strychnine is shown in Figure 3 [20,21,22,23], and MS^2^ and MS^3^ spectra of strychnine in matrix are shown in Figure 4.

The procedure was validated according to the (Guidance Document) SANTE/11945/2015 [18]. The validation parameters: linearity, repeatability, reproducibility, average recovery, selectivity, matrix effect, screening detection limit (SDL) and limit of quantification (LOQ) were evaluated. The analysis of 20 blank samples of the matrices did not reveal any interference. The criteria concerning relative retention time of the analytes corresponded to those of the calibration solution at a tolerance of ±2.5%. A linearity (R^2^) for all concentration levels (25–1000 µg/kg) was obtained and was 0.995. The screening detection and quantification limits are presented in Table 1. The apparent recoveries for all concentration levels (25–1000 µg/kg) were in the range of 98.97% to 104.0% with a repeatability less than 4.1% (2.9%–4.1%), and within-laboratory reproducibility below 4.6% (3.3%–4.6%). The expanded uncertainty was calculated at four concentration levels, between 25 and 1000 µg/kg, a factor of 2, which provided a level of confidence of approximately 95% (Table 2). The calculated ion suppression of the matrix effects for strychnine in liver did not exceed 5.2% at the level of 25 µg/kg. The screening detection and quantification limits are presented in Table 1.

The developed and validated method meets the criteria set for analytical procedures that can be used in toxicological analyses. The validation showed high repeatability and reproducibility with an average recovery of 104% for the HPLC-MS^3^ method. The use of this technique enabled the determination of strychnine with improved selectivity as compared to the method by adding an additional MS^2^ fragmentation compound. In recent years, more than 400 samples have been analyzed using the screening method [12]. Of these, nine were suspected of strychnine presence (peaks in both transitions). In three of them, suspicion was confirmed. Note that suspicious results were most frequently related to cases where there was a progressive decomposition of the materials sent for testing. In such cases, various types of contamination are more frequent and may cause disturbances in ion relationships and false-positive or false-negative results. By using the presented HPLC-MS^3^ method in one case, a suspected wolf poisoning, strychnine below the limit of determination in the liver was found; the other two cases involving dog poisonings, 123 and 150 ug/kg, respectively, were found in the liver. In the last case, additional analyses were performed in the stomach contents, where the concentration of 1694 µg/kg was determined.

## 3. Materials and Methods

### 3.1. Materials

All reagents were of minimum analytical grade or higher. Acetonitrile (ACN), acetic acid, methanol (MeOH), ethanol, isopropanol, and octadecylsilane (C18) sorbent were supplied by J.T.Baker (Deventer, the Netherlands). Ultrapure water was filtered through a Milli-Q system (Millipore, Burlington, MA, USA). Nanosep MF 0.22 μm filters were supplied by Pall (Port Washington, NY, USA). Primary-secondary amine (PSA) was purchased from Supelco (Bellefonte, PA, USA). Sodium acetate and magnesium sulphate (MgSO_4_) were purchased from POCh (Gliwice, Poland). Strychnine was obtained from Sigma-Aldrich (St. Louis, MO, USA). Carbofuran-D3 (internal standard) was supplied by Dr Ehrenstorfer (Augsburg, Germany).

### 3.2. HPLC-MS/MS/MS

The HPLC-MS^3^ system consisted of an ABSciex Exion LC HPLC system connected to ABSciex API 5500 Qtrap mass spectrometer (AB Sciex, Concord, Canada). The Analyst 1.6.3 software controlled the HPLC-MS^3^ system, and Multiquant 3.2 was used to process the data. The MS was operated as previously described with some modifications [12]. Briefly, desolvation temperature was set at 500 °C, gas 1 (air)-45 psi; gas 2 (air) – 45 psi; collision gas (N_2_)-high; nebulizer gas (N_2_)-40 psi; curtain gas (N_2_) – 25 psi. The voltage of the electron multiplier and the electrospray capillary were set at 2100 V and 5500 V, respectively. The chromatography was performed in a Kinetex^®^ 2.6 µm C8 column 50 × 2.1 mm^2^ (Phenomenex, Torrance, CA, USA), connected to a SecurityGuard™ ULTRA C8 2.1 mm precolumn (Phenomenex, USA). The mobile phase was composed of two solutions: A (0.5% isopropanol in 0.1% acetic acid in water) and B (5% isopropanol in ethanol) in a gradient mode which started with 2% of B. From 1 to 2 min, the concentration of B was raised to 70% and left for 1 min. Finally, after 1 min, the concentration of B was decreased to 2% and left for 1 min. The flow rate was 0.4 mL/min. The column was working at 30 °C. The ions were monitored in MS^3^ mode (Table 3). The mass spectrometry parameters for the internal standard were as follows: the ions monitored by MRM were 224.8→165. The declustering potential (DP) was 96 eV. The optimized collision energy (CE) for internal standard was 12 eV for the first daughter ion.

### 3.3. Sample Preparation

The extraction procedure is similar to the previously described method for diagnosing fatal poisoning cases in animals [12]. A 1 ± 0.05 g sample of homogenized liver was mixed with 2.5 mL of ACN on a vortex mixer at 349× rcf (relative centrifugal force) for 1 min. Then, 0.5 mL of 5 molar ammonium acetate solution with a pH of 7.8 was added and mixed with the same parameters applied for 1 min. The resulting suspension was sonicated for 15 min in an ultrasonic bath, mixed on a vortex mixer for 1 min, and centrifuged at 2930× rcf at room temperature for 10 min. The supernatant solution was collected and a 0.7 mL aliquot of each extract was added to a new centrifuge tube with 150 mg of MgSO_4_, 50 mg of C18, and 50 mg of PSA. The contents of the tube were mixed on a vortex mixer for 1 min and centrifuged for 10 min at room temperature at 2930× rcf. The supernatant solutions were transferred to Nanosep filters MF (0.2 μm) (Pall, Port Washington, NY, USA) and centrifuged at 9447× rcf for 10 min at room temperature before being injected into the LC column.

### 3.4. Validation

The analytical method validation was carried out according to the (Guidance Document) SANTE/11945/2015 [18]. The following validation parameters were established: selectivity, linearity, precision, SDL, and LOQ. Unfortunately, because internal standard is not available for strychnine, deuterated carbofuran was used. Some authors used other substances such as brucine or papaverine [24,25,26], but they may also appear as poison, so they were not considered.

Analyte standard solutions at different concentrations: 25, 100, 250, 1000 µg/kg were added to the blank sample containing an internal standard (100 µg/kg), subjected to the QuEChERS and HPLC procedure. The analyte peak area was plotted against the corresponding concentrations and the calibration curves were set up by means of the least-squares method.

SDL and LOQ were estimated by calculations based on signal-to-noise ratio. Determination of the signal-to-noise ratio was performed by comparing measured signals from samples with known low concentrations of analyte with those of blank samples and establishing the minimum concentration at which the analyte can be reliably detected or quantified. A typical signal-to-noise ratio is 3:1 for SDL and 10:1 for LOQ.

Spiked blank samples were prepared as follows: standard solutions of different concentration corresponding to 25, 100, 250, 1000 µg/kg and internal standards (carbofuran-D3) corresponding to 100 µg/kg were added to 1 g of homogenized liver sample. Spiked blank samples were analyzed according to the previously described procedure. The repeatability and reproducibility were determined at four concentration levels (six samples of each level): 25, 100, 250, 1000 µg/kg. For repeatability, the samples were analyzed by the same operators on the same day with the same instrument, and were calculated as the relative standard deviation (RSD, %). For within-laboratory reproducibility, two other sets of blank samples were fortified and analyzed by different operators, on two other days with the same instrument, and were calculated as the relative standard deviation.

In the selectivity study, possible interferences encountered in the method were checked by analysis of 20 blank samples for each matrix from different sources.

The recovery was calculated by comparing the mean measured concentration with the fortified concentration of the samples.

The matrix effect was checked by analyzing five different samples at 25 µg/kg concentration, which is the LOD for this method and calculated by the equation proposed previously by Matuszewski [27].

The expanded uncertainty was calculated at the four concentration levels corresponding to 25, 100, 250, 1000 µg/kg by applying a coverage factor of 2, which gives a level of confidence of approximately 95% [28].

## 4. Conclusions

To the best of our knowledge, this is the first simple method based on the QuEChERS and HPLC-MS^3^ combination reported for the determination of strychnine in liver matrices. This method is used for effective routine toxicology analysis in Poland.

## Figures and Tables

**Table 1:** Validation report for strychnine. SDL: screening detection limit.

Matrix	SDL [µg/kg]	LOQ [µg/kg]	Matrix Effect (%)	Concentration Range (ng/mL)	Determination Coefficient	Calibration Curve
Liver	2.0	25	5.2 ± 2.1	25–1000	0.995	*y* = 0.8134(±0.04)*x* + 0.012(±0.06)

**Table 2:** Parameters obtained for the calibration curve.

Level	Repeatability (RSD_r_, %) (n = 6)	Within-lab Reproducibility (RSD_wR_,%) (n = 18)	Apparent Recovery (%)	Expanded Uncertainty (µg/kg)
25 µg/kg	3.4 ± 3.1	4.6 ± 3.1	104.0 ± 2.8	25 ± 5.2
100 µg/kg	2.9 ± 3.4	4.6 ± 3.3	103.5 ± 3.8	100 ± 12.3
250 µg/kg	3.0 ± 2.7	3.3 ± 3.4	104.0 ± 4.2	250 ± 23.2
1000 µg/kg	4.1 ± 2.9	3.7 ± 3.7	98.7 ± 6.3	1000 ± 40.8

**Table 3:** Precursor ions of strychnine—mass spectrometry parameters.

Analyte	1st Precursor Ion (*m*/*z*)	2nd Precursor Ion (*m*/*z*)	MS^3^ Ion Scan (*m*/*z*)	Width (Da)	Excitation Energy (eV)	Declustering Potential (eV)	Entrance Potential (eV)	Collision Energy (eV)
Strychnine	335.2	184.0	156 129	1 1	0.1 0.1	290 290	10 10	61 61
